# Optimization of school closures during an Omicron epidemic in Hong Kong: a modelling study

**DOI:** 10.1098/rsfs.2025.0016

**Published:** 2025-09-26

**Authors:** Benjamin R. Young, Faith Ho, Yun Lin, Eric H. Y. Lau, Peng Wu, Benjamin J. Cowling, Tim K. Tsang

**Affiliations:** ^1^ The University of Hong Kong, Hong Kong, Hong Kong Special Administrative Region, Hong Kong; ^2^ Department of Community Medicine and School of Public Health, The University of Hong Kong, Hong Kong, Hong Kong; ^3^ Laboratory of Data Discovery for Health Limited, New Territories, Hong Kong Special Administrative Region, People’s Republic of China

**Keywords:** epidemiology, infectious diseases, COVID, influenza, viruses, policy

## Abstract

Closing schools has been a prominent public health control measure for respiratory virus pandemics. However, during the COVID-19 pandemic, they were more contentious, as children were at a lower risk of severe disease while prolonged closures could have affected children’s development. Hong Kong experienced a large Omicron epidemic in the spring of 2022, and face-to-face classes were halted and reopened after the peak with daily rapid-antigen test screening. Using counterfactual simulations, we developed a susceptible-exposed-infectious-recovered model calibrated to severe disease data to estimate the impact of the school closures and screening measures. We estimated that the school closures and screening measures prevented an excess of 35.0% (33.2%, 38.8%) more deaths and 17.4% (15.0%, 19.9%) more hospitalizations in a counterfactual scenario without those interventions. In terms of the impact on disease burden, the closure of primary schools outperformed both the closure of secondary schools and rapid antigen testing. Rapid-antigen screening alone was also an effective measure while minimizing the disruption associated with school closures. This demonstrates that implementing school-non-pharmaceutical intervention requires input from community priorities, balancing the population-wide burden of disease (infections, hospitalizations or mortalities) or educational disruptions (missed school days) and economic repercussions (e.g. the cost of daily rapid-antigen screening).

## 1. Introduction

During the COVID-19 pandemic, school closures emerged as a widespread yet controversial non-pharmaceutical intervention (NPI). Historically, school closures have been effective in respiratory virus pandemics, slowing transmission and protecting healthcare systems [1,2]. This effectiveness is largely attributed to high contact rates in schools, which act as amplification settings where infected children spread the virus to households and communities [3–5]. However, COVID-19 presented a more complex challenge. Unlike in previous pandemics, children had a lower risk of severe disease compared with adults [6–8], while their susceptibility to infection was uncertain [9,10]. The Omicron variant further complicated the impact of school closures, with greater transmissibility in children [10,11].

Control measures targeting children and schools can be contentious, for infections such as SARS-CoV-2 that are very rarely severe in school-age children. School closures are implemented to reduce infections not only in children and adults within the school, but also to limit transmission more broadly in the community; however the overall impact remains unclear. At the same time, schools provide critical functions beyond education, including social development, daily nutrition and healthcare access. The negative impacts of school closures are broad and largely unquantified [12–14], ranging from slower development [15,16] and exacerbated mental health issues [17,18], to widening socioeconomic inequalities [14,16,19]. Caregiver workplace absenteeism further leads to economic losses and strained healthcare sectors [20,21]. Global responses to school closures varied widely over the pandemic, shaped by resources, cultural priorities and pandemic strategies, amid uncertainty about the effectiveness and impact of school-based NPIs [20,22].

Determining the effectiveness of school-based NPIs is essential for helping decision-makers balance disease control efforts with societal costs. However, the effectiveness of interventions like school closures and rapid antigen testing (RAT) programmes varies across studies [22–24], partly due to challenges such as concurrent NPIs, observational study limitations and dynamic pandemic factors. Community-wide NPIs and self-initiated behavioural changes complicate isolating the effects of specific interventions, while temporal and spatial variation in variants and unique epidemic histories limit generalisable conclusions. Age-varying disease risk profiles may further bias the assessment of children’s contribution to transmission. Mathematical models offset these limitations, allowing the simulation of underlying epidemic curves and counterfactual scenarios and estimating the effect of individual interventions.

Hong Kong’s first Omicron wave, beginning in January 2022, provides a case study for evaluating the effectiveness of school-based NPIs. During this period, a 13−15 weeks school disruption occurred from mid-January to mid-April, varying by school type [21,25]. Initially, face-to-face classes were suspended, later transitioning into an early summer holiday. This closure, followed by mandatory RAT screening upon resumption, coincided with an unprecedented wave of over 50 000 daily confirmed cases and a high mortality rate among older adults [26], with over half the population infected [27].

This study uses mathematical modelling of observed and counterfactual scenarios to evaluate the impact of school NPIs during Hong Kong’s Omicron wave and identify optimal implementation strategies. Calibrated to severe disease data, which are more robust to variability in case ascertainment [28], our models quantify the effects of school closures on transmission and severe disease outcomes. By assessing prevalence-based NPI triggering thresholds, it compares interventions across primary and secondary schools, including daily RAT screening. Optimal strategies are determined by evaluating trade-offs between disease control benefits and societal repercussions, such as missed school days and associated costs.

## 2. Methods

### 2.1. Model structure

We developed a susceptible-exposed-infectious-recovered (SEIR) compartmental model to simulate Hong Kong’s BA.2-dominated Omicron wave between 1 January and 30 April 2024. The Hong Kong population of 7.4 million comprises 6.7% of kindergarten/primary school-aged children (3–11 years) and 4.5% of secondary school-aged children (12–17 years) [29]. The model structure has been previously described [30]. The model used an age- and dose-structured framework with 10 age bands and four vaccination levels to estimate age- and dose-specific risks of hospitalization, severe disease and mortality.

The force of infection ($\lambda_{t}$) is determined by the age- dose-specific transmission parameter ($\beta_{iv}$), and the total number of infectious individuals over time t, stratified by symptomatic and asymptomatic infections (electronic supplementary material, appendix). The age- and dose-specific transmission parameter combines the time-varying contacts ($C_{ij}\left(t\right)$) and age- and dose-specific relative transmissibility parameters. Contact matrices were based on a pre-COVID Hong Kong social contact survey [31], adjusted over time for behavioural changes and public health measures using Google mobility data and government-reported school closures, assuming linear relationships between mobility and contact rates across settings, as detailed in electronic supplementary material appendix [32,33]. Transmissibility parameters included the probability of infection per contact, estimated through model fitting, the symptomatic [34], age- and dose-specific relative infectiousness and susceptibility [9,35,36] derived from the literature (electronic supplementary material, appendix). Face-to-face school suspensions or closures were accounted for by setting school-specific contacts to zero. Daily RAT screening in schools was incorporated by scaling school-based contacts by the mean test sensitivity (53%) of licensed tests in Hong Kong at the time [37] (electronic supplementary material, appendix). Negative tests were required for school attendance [25].

### 2.2. Model fitting

The model was fitted using a Bayesian framework, as described previously (electronic supplementary material, appendix and [30]). Severe disease incidence (hospitalizations, severe or critical cases (SCC) and deaths) was estimated by convolving the SEIR-model predicted infection incidence with age- and dose-specific risks of severe events and delay distributions (electronic supplementary material, appendix). The model-expected severe incidence data realized a negative binomial distribution and was aggregated into five broader age groups, to ensure sufficient events for calibration to observed severe disease incidence data. Calibration was performed using Markov Chain Monte Carlo simulations with the Metropolis–Hastings algorithm. The observed data included individual-level hospitalization records with vaccine doses, admissions, SCC, deaths and dates of symptom onset, reported cases and severe events, aggregated by age and dose, corresponding to the simulated data. The hospital authority of Hong Kong initially provided the hospitalization data; informed consent was omitted as the data was part of the emergency authorization.

We accounted for age-varying risks of severe disease during the peak period of the Omicron epidemic. Outside of the peak period, the age- and dose-specific risks of severe events $(p_{\psi ,i,v}$) were assumed to remain constant. During the peak period daily excess hospitalizations ($H_{excess}$) were calculated as the difference between the rolling 7 day mean of hospitalizations and a predefined threshold ($H_{excess}$ = $H_{roll}-H_{threshold}$). An age-specific modifier (δ) adjusted the risk of severe disease based on a fitted linear relationship, defined as:


$$\delta =\left\{ \begin{array}{rl} 1, & \text{ if }H_{\text{excess }}\leq 0\\ 1+\beta_{1}\cdot H_{\text{excess }}, & \text{ if }0<H_{\text{excess }}\leq H_{\text{excess }}^{\max}\\ 1+\beta_{1}\cdot H_{\text{excess }}^{\max}, & \text{ if }H_{\text{excess }}>H_{\text{excess }}^{\max} \end{array}\right.$$

The slope of the linear relationship (*β₁*) was determined during model fitting to calibrate to the observed age-specific severe outcomes during the epidemic peak. $H_{excess}^{max}$ was set to the maximum value corresponding to the observed peak hospitalizations as the relationship between excess hospitalizations and modified risk beyond the observed data is uncertain. The age- and dose-specific outcome risk is modified as:


$$p_{adj,\psi ,i,v}=p_{\psi ,i,v}\cdot \delta_{i}.$$

This approach enabled a more nuanced estimation of severe outcome-modified risks for counterfactual simulations during peak periods. The model accounts for the benefits of flattening the curve or increased risks under surges.

### 2.3. Simulations

Three simulations were run: (i) the observed scenario with actual NPI timing, (ii) a no-intervention counterfactual, and (iii) counterfactual scenarios exploring a range of NPI-timing using prevalence thresholds ($\theta_{p}$, $\theta_{s}$, $\theta_{r}$) for primary and secondary school closures and daily RAT screening, respectively (table 1 and electronic supplementary material, appendix). For school closures, prevalence was calculated within respective populations ($I_{p}/N_{p}$ for primary, $I_{s}/N_{s}$ for secondary). Daily RAT implementation was based on the community-wide prevalence (I/N). The threshold prevalence triggers ranged from 0.001 (early/strict) to ∞ (never). To simulate the school closures, $C_{ij}$ were modified during interventions according to:


$$C_{ij}=\;C_{ij,not\;schools}+\left\{ \begin{array}{ll} \;0C_{ij;school,p}\; & \;if\;I_{p}/N_{p}\geq \theta_{p}\\ 0C_{ij;school,s}\; & \;if\;I_{s}/N_{s}\geq \theta_{s}\\ \;\alpha C_{ij;school}\; & \;if\;I/N\geq \theta_{r} \end{array}\right..$$

**Table 1.** The NPI repercussions associated with each of the simulated prevalence thresholds, no-intervention and observed scenarios, across the school-related NPIs, primary and secondary school closures and RAT daily screening.

simulation scenario	NPI	initiation prevalence (%)	school intervention impact	
			face-to-face days disrupted	days with daily RATs
observed interventions	primary schools	—	64	8
	secondary schools	—	62	0
no interventions	all	—	0.0	0.0
initiate NPI	primary schools	0.1	83	17
		2	43	17
		4	24	22
		6	10	36
		∞	0	46
	secondary schools	0.1	84	7
		2	40	6
		4	12	22
		∞	0	35
	daily RATs	0.1	30	144
		2	37	61
		4	39	22
		6	42	3
		∞	53	0

### 2.4. Statistical analysis

Disease burden assessment compared outcomes across simulation scenarios, including infections, hospitalizations, deaths and years of life lost (YLL, calculated using Hong Kong life expectancy data [38]). NPI-related repercussions, such as days of RAT implementation and total school days missed (for primary and secondary schools) were also recorded. Results were evaluated against the no-intervention scenario to quantify absolute and relative changes in disease burden and school-related disruption.

The relationship between school-NPI prevalence thresholds and disease outcomes was analysed using generalized additive models (GAM) with and without interactions, using smoothing splines to capture nonlinearity. Models incorporating two- and three-way interactions used tensor product smooths (see electronic supplementary material, appendix for model selection). Significant interactions were summarized through stratified effects, presented as absolute and relative differences, while partial dependency plots showed effects across thresholds, holding other NPIs constant.

To translate findings into policy-relevant insights, we developed an optimization algorithm to identify optimal NPI-prevalence trigger combinations. The algorithm minimized a weighted composite score of cumulative outcomes, including missed school days and RAT implementation days, with weights (0–1 in 0.1 increments) reflecting policy priorities. Standardized outcomes were combined to identify threshold settings (*θ*) that minimized the weighted sum. Ternary plots illustrated the optimal NPI thresholds across priority weightings for deaths, infections and combined NPI repercussions (school closures and RAT days). For the latter parameter, school closures and RAT days were weighted 8 : 2, reflecting an emphasis on minimizing educational disruption and the practical use of RAT screening in line with local preferences [39]. Sensitivity analyses tested variations in disease metrics, weighting NPI-repercussions and outer bounds of transmissibility parameters. All analyses were conducted using R version 4.2.3.

## 3. Results

Hong Kong’s Omicron wave (January–April 2022) resulted in 8434 deaths and 51 440 hospitalizations. Schools were closed for most of the semester, with face-to-face days suspended or fully closed for 83% (64 days) and 81% (62 days) of primary and secondary school days (table 1). During this period, there were eight days of mandatory school-wide RATs in primary schools (table 1). The fitted compartmental model simulating the observed school closures scenario produced estimates of 8438 deaths (95% CrI: 8050, 8853) and 52 239 hospitalizations (95% CrI: 51 073, 53 572), respectively (electronic supplementary material, appendix). The estimated population attack rate was 55.1% (4 081 527 [3 978 866, 4 197 407]).

**Figure 1.**
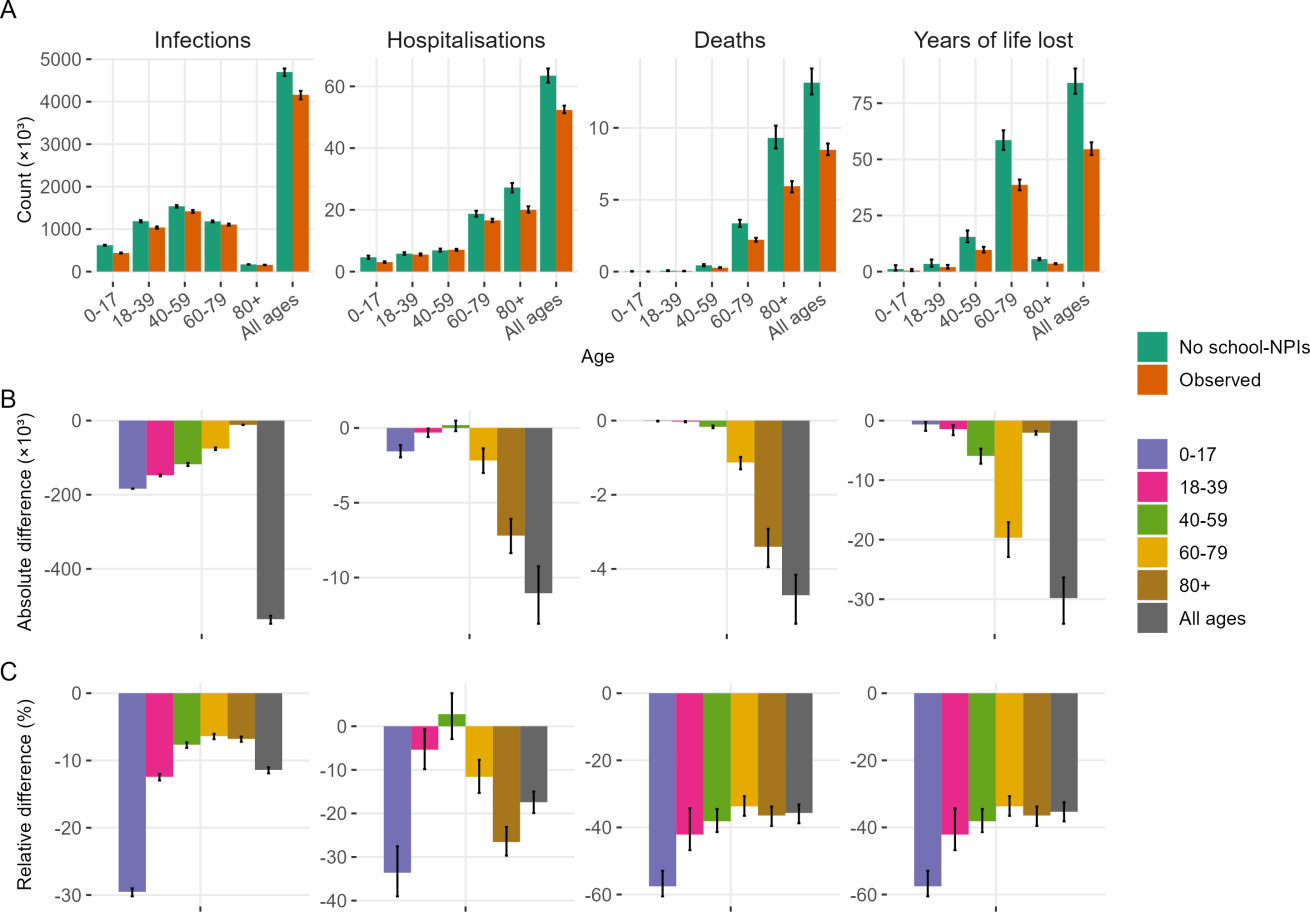
Based on the fitted model, (A) the total impact across ages for the observed scenario and no-intervention simulation is shown. The (B) absolute and (C) relative differences are also shown across ages.

The age distribution of severe outcomes was predominantly driven by elderly risk, while infections and hospitalizations were more dependent on population contact patterns (figure 1A). Estimated disease risk increased with age, showing a steeper gradient for more severe outcomes (electronic supplementary material, appendix). During the peak period, severe outcome risk displayed age-dependent amplification with hospitalization showing a characteristic J-shaped curve and decreased risk in middle-aged groups (electronic supplementary material, appendix). Adults aged over 80 years accounted for approximately 40% of hospitalizations and 70% of deaths, while years of life lost (YLL) peaked in the 60−79 age group (figure 1A). The age distribution of total infections mirrored population mixing patterns, with a distinct peak in middle-aged groups.

### 3.1. No school closure versus observed

**Figure 2.**
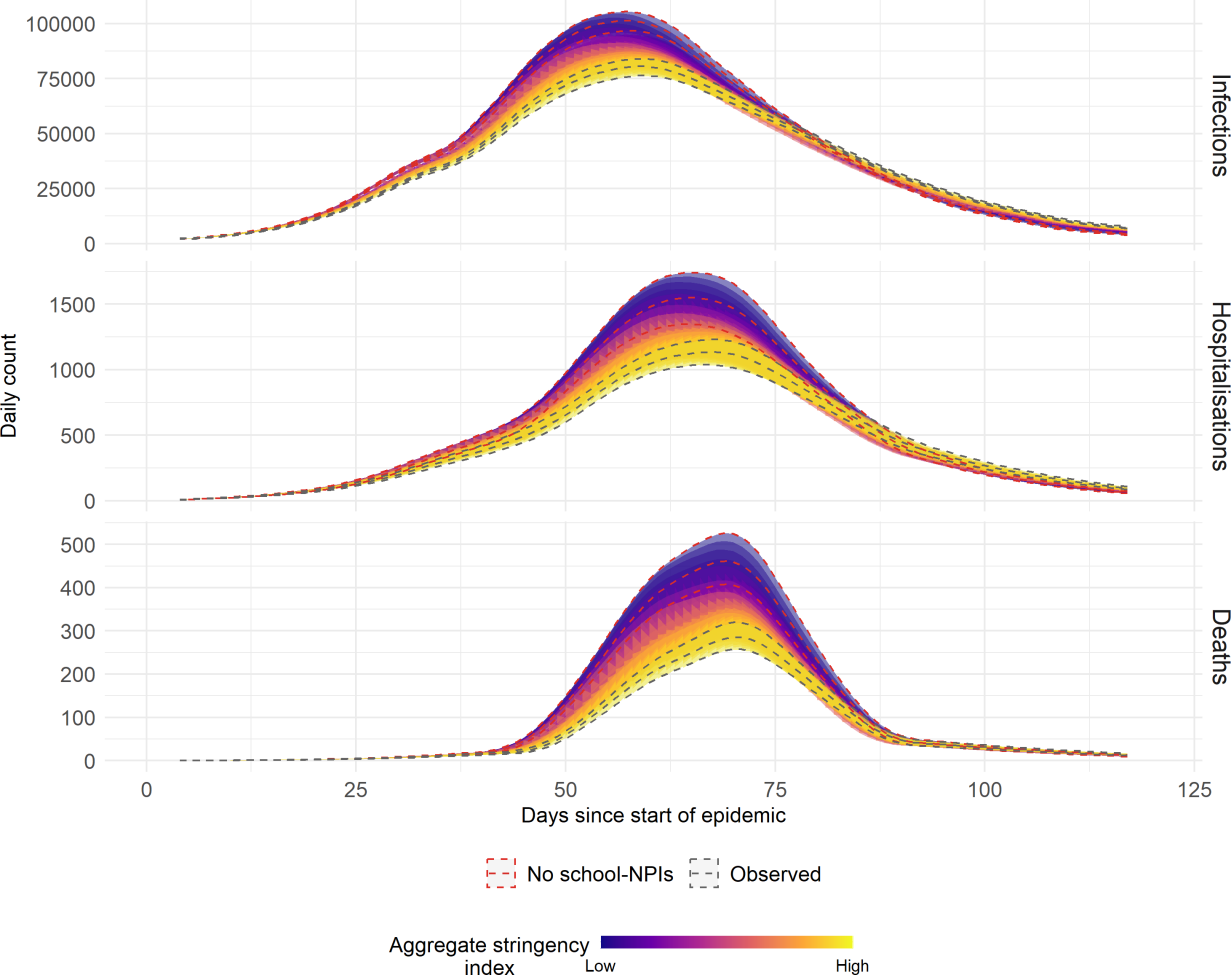
The time-series severe events over time based on the rolling mean over 7 days, for the no-intervention scenario (baseline), the observed NPI scenario and the gradient represent the prevalence-threshold NPI initiation simulations, where high stringency is all implemented at 0.1% and low stringency, never implemented.

The no-intervention scenarios showed a significant increase in disease burden. The model estimated 13 143 deaths (95% CrI: 12 327, 14 130), 63 465 hospitalizations (95% CrI: 61 197, 65 780) and 4 698 592 infections (95% CrI: 4 602 818, 4 781 532) in the absence of school closures (figure 1A). School closures had the strongest effect on mortality, reducing deaths by 35.0% (33.2%, 38.8%), hospitalizations were decreased by 17.4% (15.0%, 19.9%), while infection reduction was more modest at 11.4% (11.0%, 11.9%) (figure 1B). The impact on YLL paralleled mortality reductions but showed age-weighted variations. The implemented interventions not only reduced the magnitude but also delayed peak outcomes for infections, hospitalizations and deaths (figure 2).

The age-specific impacts of school closures varied by outcome (figure 1B,C). Hospitalization reductions showed a ‘U’-shaped distribution, with the largest reductions in children (−33.6% [−39.0%, −27.5%]) and adults over 80 (−26.6% [−29.7%, −23.1%]), respectively, reflecting the direct beneficiaries and the most vulnerable group. Middle-aged adults (40−59-year-olds) showed no significant change in hospitalizations (2.8% [−3.0%, 7.6%]), possibly due to reduced peak-period risk. Individuals under 18 experienced the greatest relative benefits across most outcomes but with small absolute differences; however, infections had both maximum relative and absolute reductions. Among adults over 18 years (indirect beneficiaries), interventions produced uniform relative impacts across infections, deaths and YLL, with a slightly higher impact in 18−39-year-olds, likely due to greater contact with school-aged groups (electronic supplementary material, appendix). The oldest age groups received the greatest absolute benefit in severe outcomes, while YLL reductions peaked in the 60−79 age group. The age-related impact of school closures and daily RATs ultimately reflected three key factors: age-specific risk, peak-period risk amplification and contact patterns.

### 3.2. Closures initiated on prevalence triggers

The disease outcomes varied with the stringency of prevalence-trigger thresholds for school NPIs (figure 2). Across all three NPIs (primary, secondary closures and daily NPIs) a combination of the most stringent ($\theta_{i}=0.001$) achieved outcomes similar to the observed school closures, while no initiation ($\theta_{i}=\infty$) produced outcomes equivalent to the no-intervention scenario. Lower prevalence thresholds progressively flattened and delayed outcome incidence curves. School days missed scaled directly with stringency, ranging from 0 to 145 days for both primary and secondary schools. The most stringent scenario reached maximum closure days as prevalence remained above 0.1% throughout the study period (table 1).

**Figure 3.**
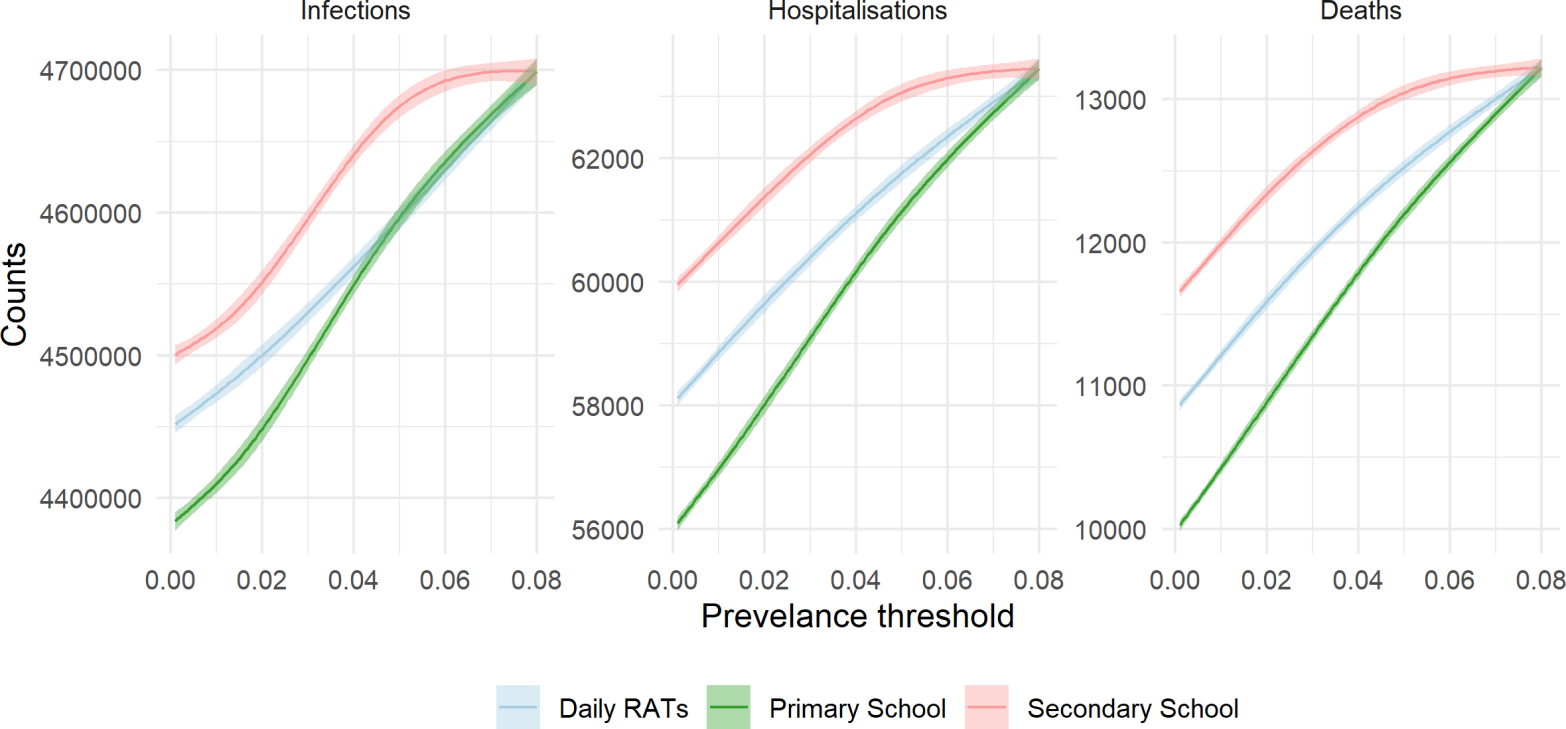
Partial dependency plots for the effect of each school NPI (primary and secondary school closures and RAT daily screening) on severe outcomes. Plots are based on the fitted GAM model, where the other NPIs are assumed not to be initiated.

**Figure 4.**
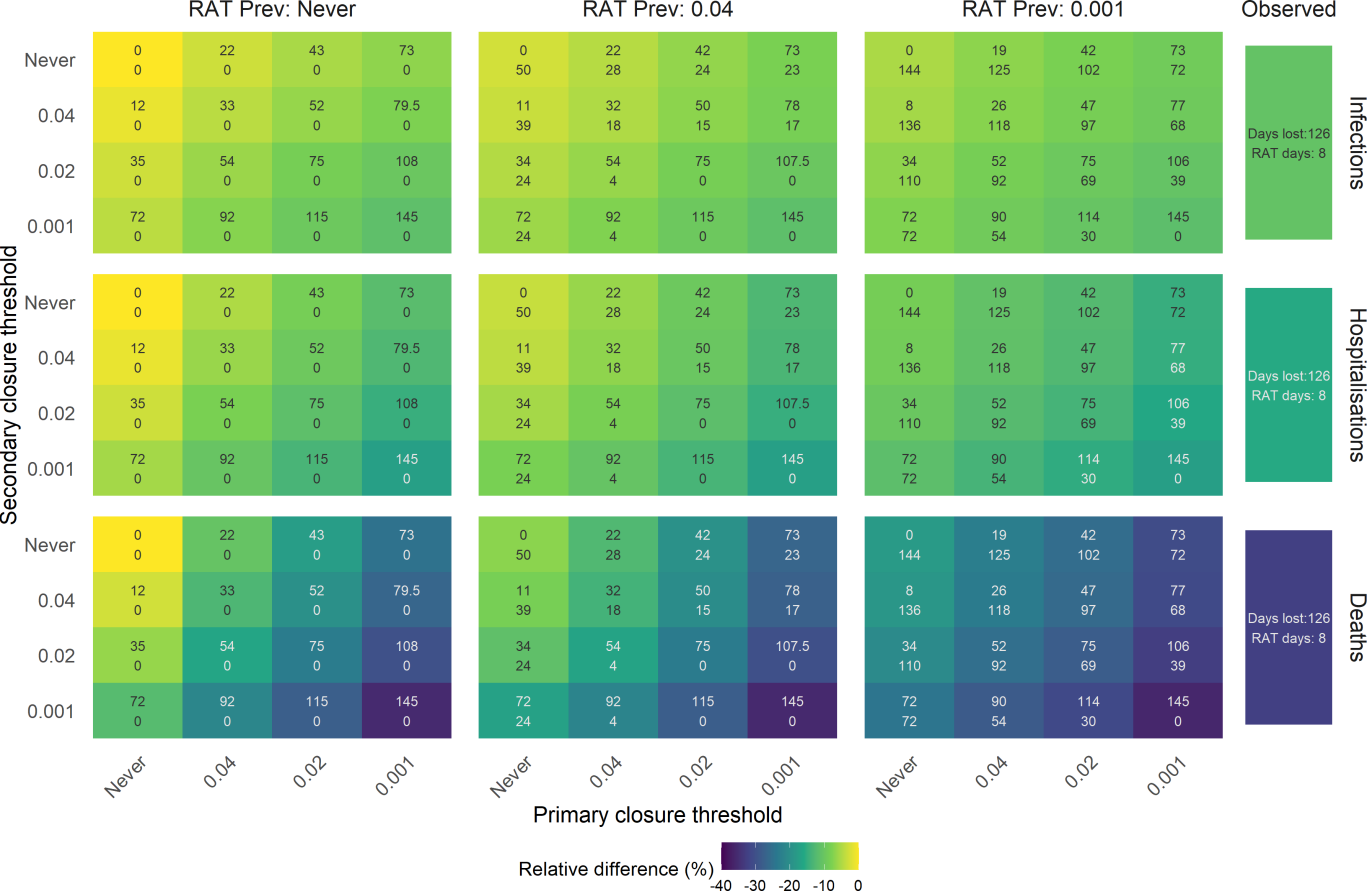
Stratified effects of the prevalence-threshold simulations for the school-NPIs (primary and secondary school closures and RAT daily screening) and observed NPI scenarios. Relative differences are calculated from the no-intervention. Within the tile, values represent the associated school days missed and the total days with RATs across primary and secondary schools.

Primary school closures demonstrated the most substantial effects across all outcomes, although the effectiveness of each school intervention interacted significantly with concurrent interventions. GAMs with splines analysed the relationship between the prevalence threshold for school-NPIs and outcomes (infections, hospitalizations, deaths or YLL). Significant three-way interactions were identified across all outcomes (*p* < 0.001, electronic supplementary material, appendix). Partial dependency plots were used to examine the marginal effects of each school-NPI while holding others inactive (θ = ∞ ) (figure 3). Primary school closures had the strongest impact per 1% change in threshold across all outcomes, followed by daily RAT screening. Secondary school closures were demonstrated to be the least effective (figure 3). Given the significant interactions between the three school-NPIs, stratified results across all combinations are presented in figure 4.

Stratified prevalence-threshold simulations demonstrate how combinations of school-related NPIs can achieve substantial disease prevention (figure 4). Daily RAT screening alone, at maximum stringency ($\theta_{p,s}=\;\infty \;and\;\theta_{r}=0.1\%$), achieved approximately half of the impact of the observed scenario, reducing deaths by 18.3% (95% CI: 16.9%, 20.2%), hospitalizations by 8.7% (7.3%, 10.2%) and infections by 5.3% (5.1%, 5.7%) (figure 4). Strict primary school closures and daily RATs, without closing secondary schools ($\theta_{p,r}=0.1\%\;and\;\theta_{s}=\infty$), reduced deaths by 30.7% (28.2%, 33.6%), hospitalizations by 14.9% (12.7%, 17.1%) and infections by 9.3% (8.9%, 9.7%) infections (figure 4). These reductions account for approximately 85–90% of averted outcomes as the observed scenario, with 72 primary face-to-face days missed and no secondary school days disrupted. While the observed scenario averted a substantial disease burden, stratified results reveal that primary school closures and daily RATs alone can achieve a significant proportion of this prevention.

### 3.3. Optimizing school non-pharmaceutical intervention

**Figure 5.**
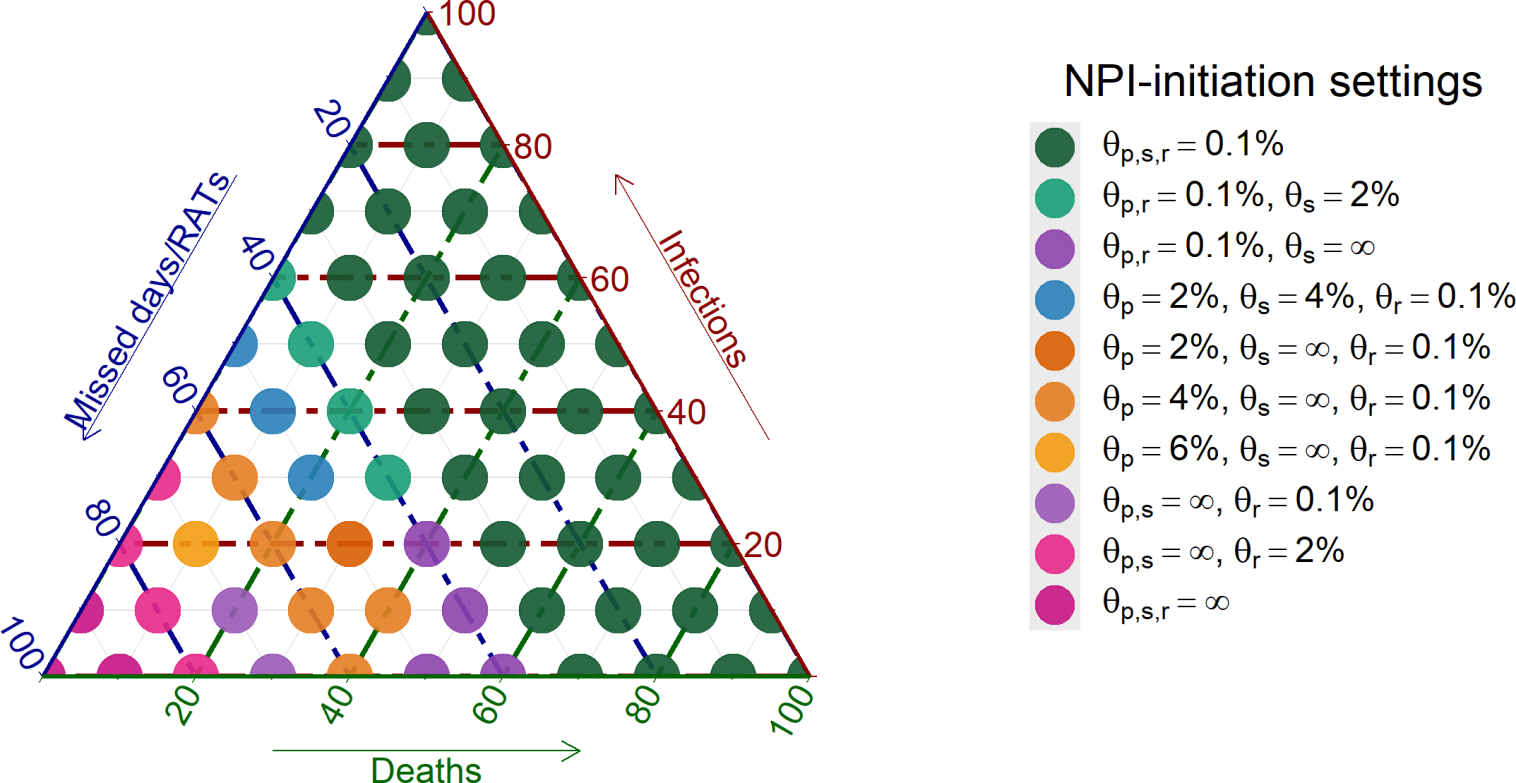
Ternary plot to show the preferred set of prevalence thresholds which initiate the respective NPI (primary and secondary school closures and RAT daily screening), based on the weighting of the outcomes (infections, deaths and missed days/days with RATs, weighted 8:2).

**Figure 6.**
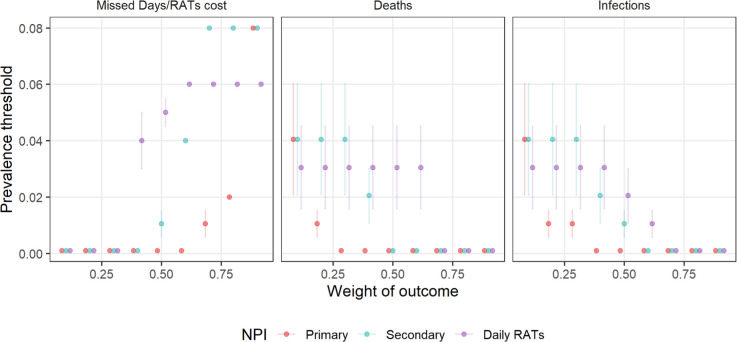
Average NPI prevalence thresholds are based on the weighted outcomes across three key metrics: NPI impacts, deaths and infections. Thresholds are shown for primary and secondary school closures and daily RAT screenings.

A clear trade-off exists between preventing disease and minimizing negative repercussions, such as missed school days or the cost of RATs (figure 5). We illustrated the optimal threshold settings by balancing population-wide disease metrics (infections and deaths) with NPI repercussions (missed school days and daily RAT costs, weighted 8:2). The optimization model revealed three distinct recommendation patterns. For high disease control priority (>60%), the model recommends the most stringent thresholds ($\theta_{all}=0.001$), for all three school-NPIs. Under balanced priorities (disease metrics combine to 40–60%), the model increasingly favours keeping schools open while using RATs to mitigate disease. When minimal disruption is prioritized (disease control <40%), the model recommends only daily RAT screening and progressively increasing its threshold. A clear hierarchical pattern emerges as the priority for disease control increases (figure 6). Daily RAT screening is initiated first to suppress transmission and maintain in-person education. This is followed by primary school closures, which become progressively more stringent as the disease control priority increases. Secondary school closures are implemented last due to their lower marginal effect benefit-to-cost. This sequence reflects the relative effectiveness and disruption costs of each intervention (figures 5 and 6).

School-NPI recommendations are highly sensitive to the prioritized disease metrics. At 40% priority for minimizing repercussions, a tipping point emerges for secondary school closures. When infection reduction is prioritized over deaths, the algorithm favours closing secondary schools; when death reduction takes precedence, schools are recommended to remain open. This reflects the age distribution of benefits: secondary school closures reduce infections among children but yield limited benefits for death reduction and weaker downstream effects on older, high-risk populations. Substituting YLL for deaths (electronic supplementary material, appendix) requires a higher disease control priority before initiating closures. Similarly, combining hospitalizations and deaths, closures also require a higher threshold, as benefits to high-risk groups are small and less directly tied to contact reductions (electronic supplementary material, appendix). These metric-dependent variations in optimal intervention strategies emphasize the importance of clearly defined public health objectives when designing school-based intervention policies.

## 4. Discussion

We developed an SEIR model with age- and setting-specific contact matrices calibrated to severe disease data to evaluate the impact of school closures and daily RAT screening during Hong Kong’s Omicron epidemic in spring 2022. The implemented school-NPIs were highly effective in reducing disease outcomes, with primary school closures having the greatest impact and daily RAT screening mitigating transmission while preserving in-person education. The optimal school-NPI strategy depends on local priorities and the trade-off between disease control and societal repercussions.

Our model showed that school closures during a 13−15 week intervention reduced deaths and hospitalizations by 55 and 21%, respectively, with the greatest benefits among individuals over 60. The effects of school closures have been highly heterogeneous. Our findings align with studies showing closures are most effective during periods of high transmission, with reported reductions in mortality and incidence ranging from 0 to 60% [21,22,40–42], contrasting with studies on less contagious variants reporting only modest reduction in mortalities [11,23]. Importantly, these findings are supported by a Hong Kong-based study conducted over the same period, which found that school closures and holidays reduced incidence rates in school-aged children, and identified a higher infection risk in children relative to adults [21]. A US-wide cohort study also found greater resurgences after school reopening in high-prevalence communities or those with limited concurrent NPIs [42]. Notably, our model showed no initial resurgence when schools reopened during low prevalence with daily RAT screening in place. The heterogeneity in school closure impacts reflects contextual factors, including baseline prevalence [40], population mixing patterns [22], demographics, immunity levels and concurrent NPIs [22], and importantly, the comparative scenario [40,42]. In Hong Kong, closures were particularly effective during the severe epidemic wave, flattening the curve and limiting the hospitalization surge, which increased fatality rates up to sixfold, as observed in other studies [43,44]. Delaying the epidemic peak also amplified the benefits of an aggressive vaccination campaign, which peaked at 1378 daily doses per 1 00 000 mid-outbreak. The combined effects of transmission reduction, shorter healthcare surges and increased vaccinations provided significant mortality-reducing benefits for the older population.

Primary school closures had a greater impact on reducing transmission than secondary school closures, likely due to higher daily contact rates (2.3 versus 1.2 per student), larger populations (500 000 versus 330 000) and lower booster coverage (<0.1% versus 15%). The increased risk of unvaccinated school-aged children during Omicron outbreaks has been documented [24], and higher pre-Omicron incidence in Norwegian primary schools was attributed to greater contact rates [45]. However, evidence on age-specific transmission patterns in children remains mixed [46–48] and most studies lack the power to distinguish between primary and secondary school closures [22]. Daily RAT screening, despite low test sensitivity (53%), averted 51% of mortality alone and 86% when combined with primary school closures, largely due to daily implementation across over 8 00 000 students and staff. However, costs were significant, ranging from 10 to 100 HKD (1.3−12.8 USD) per test [49]. Research shows that testing frequency and speed of reporting outweigh sensitivity concerns [50], and while screening can minimize missed school days [24], its effectiveness varies depending on adherence, frequency and community transmission levels [51–54]. Overall, primary school closures had the greatest epidemiological impact, RAT screening was effective but costly and secondary school closures were less impactful, likely due to higher adherence to in-school NPIs among adolescents.

Our study showed that defining optimal strategies for school-NPIs depends on balancing priorities between disease control and educational continuity. When minimizing mortality, the model recommended implementing all school-based NPIs at the most stringent thresholds. While balancing disease control with educational continuity, a hierarchical approach was preferred: daily RATs were implemented first, followed by primary and secondary school closures. This reflects the differing effectiveness and costs of interventions. Studies show that tiered or part-time school reopening, combined with concurrent screening, can effectively suppress transmission [24,55,56]. These findings reflect global policy variations. By early 2021, schools averaged 6 months of closures, although with significant heterogeneity [20]. For example, Hong Kong’s prolonged closures were supported by online learning infrastructure, flexible academic calendars and domestic helpers [57], enabling a stronger focus on disease control, while Sweden prioritized educational continuity by keeping primary schools open alongside in-school mitigation measures [58,59]. Low-income regions, with limited NPI resources and larger school-aged populations, faced the longest closures globally [20,55]. These variations demonstrate that school-NPI strategies are shaped not only by epidemiological goals but also by local resources, infrastructure and societal preferences [12,13,55,59,60]. Our findings offer a framework for navigating these trade-offs, helping policymakers tailor NPI strategies to specific contexts and community priorities.

These findings have several limitations. First, while the risk of severe disease was calibrated to observed data, assumptions about age, dose and symptomatic-related infectiousness relied on prior literature. Sensitivity analyses using the outer limits of these estimates showed modest changes in effect size, but overall impact patterns and significance remained consistent. Second, simplifying assumptions about contact patterns were necessary for model identifiability, including uniform infection probabilities across settings despite behavioural differences and within settings despite age-differential NPI adherence. Similarly, nuances in school-based NPIs between school types were unaccounted for. While we assumed a uniform RAT test-sensitivity, due to limited evidence of age-related differences [61], administration for younger children places an additional burden on parents and caregivers, an omitted repercussion. Furthermore, simulations of school closures also did not account for increases in non-school contacts due to behavioural uncertainty and public space restrictions. While the model recovered observed disease outcomes, these nuances were not addressed.

Finally, simulating counterfactual scenarios is inherently challenging due to unobserved behavioural changes. For example, despite already high compliance and enforcement of public health measures [62,63], a more severe epidemic surge could introduce self-limiting behaviours, which in our no-school-NPIs scenario, may lead to an overestimating the epidemic curve and exaggerate the impact of school closures. For more conservative estimates, the model restricted peak severe disease amplification to observed hospital surge levels, although actual impacts may have been greater.

Hong Kong’s school closure strategy effectively reduced severe disease outcomes, particularly among older populations, by mitigating healthcare surges and supporting vaccination efforts. Primary school measures had the greatest impact due to differences in demographics, contact patterns and vaccination rates. While these findings are most generalizable to settings with similar characteristics, the framework for school-NPI implementation is adaptable to diverse contexts, as it accounts for local priorities and trade-offs. For instance, in settings with fewer or lower disease risk elderly populations, stricter prioritization of disease minimization may be required to initiate school closures.

Ultimately, this study highlights that community priorities shaped the optimal intervention packages. Prioritizing disease minimization led to stricter NPIs. Conversely, as priorities emphasized educational continuity, strategies shifted to delayed school closures. Although focused on Hong Kong, these findings offer broader insights into implementing school NPIs and managing the trade-offs of closures.

## Data Availability

The hospitalization data was initially provided by the Hospital Authority of Hong Kong. Due to data privacy and access regulations, we are not authorized to share this data. Associated scripts and simulated datasets are available at: https://github.com/Jamin-R12/HK-school-modelling-paper. Supplementary material is available online [64].
